# 2-[(*E*)-(2,4-Dihy­droxy­benzyl­idene)aza­nium­yl]-3-phenyl­propano­ate

**DOI:** 10.1107/S1600536811016655

**Published:** 2011-05-07

**Authors:** Hadariah Bahron, Fatimatuzzahraa Mohd Fadzel, Karimah Kassim, Madhukar Hemamalini, Hoong-Kun Fun

**Affiliations:** a Faculty of Applied Sciences, Universiti Teknologi MARA, 40450 Shah Alam, Selangor, Malaysia; b X-ray Crystallography Unit, School of Physics, Universiti Sains Malaysia, 11800 USM, Penang, Malaysia

## Abstract

The title compound, C_16_H_15_NO_4_, exists as a zwitterion in the solid state, with the carb­oxy­lic acid group being deprotonated and the imine N atom being protonated. The mol­ecule adopts an *E* configuration about the C=N double bond. The dihedral angle between the benzene rings is 46.34 (4)°. An intra­molecular N—H⋯O hydrogen bond generates an *S*(6) ring motif. In the crystal, adjacent mol­ecules are connected by inter­molecular O—H⋯O and C—H⋯O hydrogen bonds, forming supra­molecular ribbons along the *a* axis.

## Related literature

For details of Schiff bases and their applications, see: Dolaz *et al.* (2009 ▶); Kumar *et al.* (2010 ▶); Qiao *et al.* (2011 ▶); Sauri *et al.* (2009 ▶); Tamami & Ghasemi (2011 ▶). For related structures, see: Bahron *et al.* (2010 ▶); Hemamalini & Fun (2011 ▶). For the stability of the temperature controller used in the data collection, see: Cosier & Glazer (1986 ▶). For graph-set notation, see: Bernstein *et al.* (1995 ▶). 
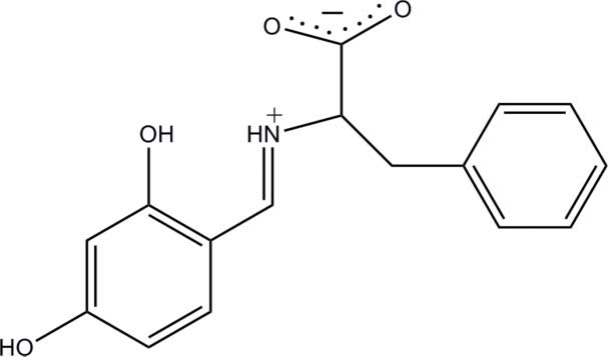


## Experimental

### 

#### Crystal data

C_16_H_15_NO_4_
*M* *_r_* = 285.29
Monoclinic, 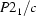

*a* = 9.3943 (1) Å
*b* = 6.8946 (1) Å
*c* = 20.7251 (3) Å
β = 92.065 (1)°
*V* = 1341.49 (3) Å^3^
*Z* = 4
Mo *K*α radiation
μ = 0.10 mm^−1^
*T* = 100 K
0.34 × 0.27 × 0.17 mm

#### Data collection

Bruker SMART APEXII CCD area-detector diffractometer
Absorption correction: multi-scan (*SADABS*; Bruker, 2009 ▶) *T*_min_ = 0.966, *T*_max_ = 0.982
22479 measured reflections
6007 independent reflections
4783 reflections with *I* > 2σ(*I*)
*R*_int_ = 0.025

#### Refinement

*R*[*F* ^2^ > 2σ(*F* ^2^)] = 0.043
*wR*(*F* ^2^) = 0.125
*S* = 1.03
6007 reflections
202 parameters
H atoms treated by a mixture of independent and constrained refinement
Δρ_max_ = 0.56 e Å^−3^
Δρ_min_ = −0.24 e Å^−3^

### 

Data collection: *APEX2* (Bruker, 2009 ▶); cell refinement: *SAINT* (Bruker, 2009 ▶); data reduction: *SAINT*; program(s) used to solve structure: *SHELXTL* (Sheldrick, 2008 ▶); program(s) used to refine structure: *SHELXTL*; molecular graphics: *SHELXTL*; software used to prepare material for publication: *SHELXTL* and *PLATON* (Spek, 2009 ▶).

## Supplementary Material

Crystal structure: contains datablocks global, I. DOI: 10.1107/S1600536811016655/rz2592sup1.cif

Structure factors: contains datablocks I. DOI: 10.1107/S1600536811016655/rz2592Isup2.hkl

Supplementary material file. DOI: 10.1107/S1600536811016655/rz2592Isup3.cml

Additional supplementary materials:  crystallographic information; 3D view; checkCIF report

## Figures and Tables

**Table 1:** Hydrogen-bond geometry (Å, °)

*D*—H⋯*A*	*D*—H	H⋯*A*	*D*⋯*A*	*D*—H⋯*A*
O1—H1*O*1⋯O4^i^	0.863 (15)	1.790 (14)	2.6302 (9)	163.9 (15)
N1—H1*N*1⋯O1	0.864 (14)	2.051 (15)	2.6810 (9)	129.1 (12)
O2—H1*O*2⋯O3^ii^	1.03 (2)	1.51 (2)	2.5310 (10)	179 (2)
C5—H5*A*⋯O2^iii^	0.95	2.56	3.3155 (10)	137
C12—H12*A*⋯O2^iv^	0.95	2.46	3.1781 (10)	132

Symmetry codes: (i) 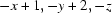; (ii) 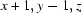; (iii) 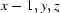; (iv) 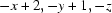.

**Fig. 1.**
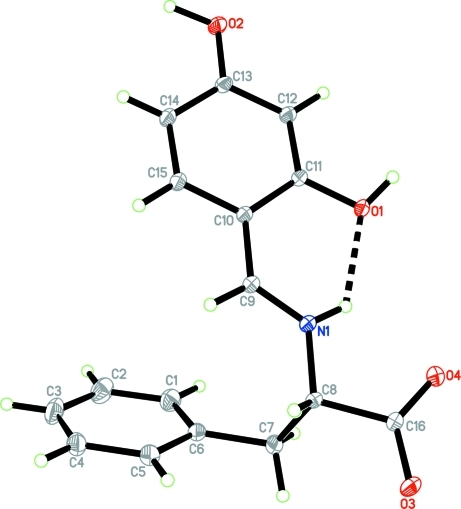
The asymmetric unit of the title compound, showing 50% probability displacement ellipsoids. The intramolecular hydrogen bond is shown as a dashed line.

**Fig. 2.**
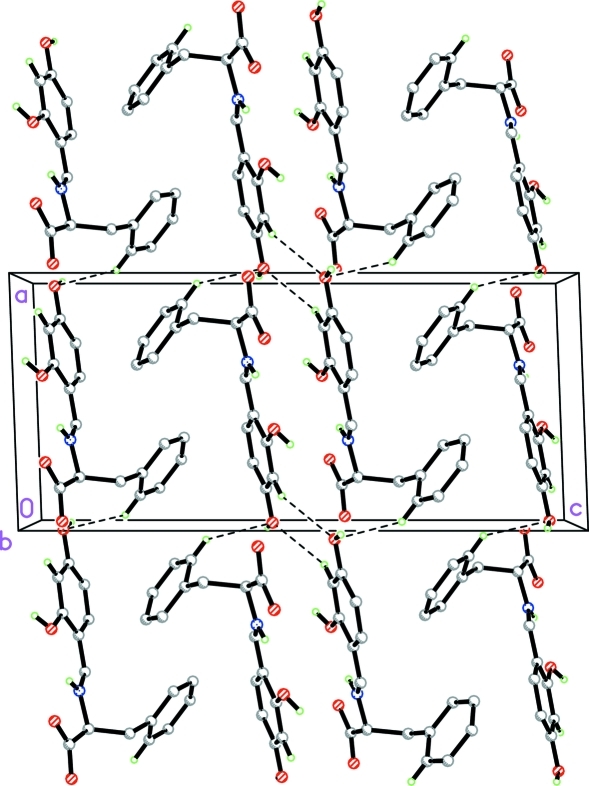
The crystal packing of the title compound with hydrogen bonds shown as dashed lines. H atoms not involved in the intermolecular interactions have been omitted for clarity.

## supplementary crystallographic information

### Comment

Schiff bases have received considerable attention and remain important in the field of coordination chemistry vastly due to their simple synthesis and versatility.  They are widely reported to have extensive potential applications in various biological and pharmaceutical fields (Dolaz *et al.*, 2009)  as antimicrobial (Kumar *et al.*, 2010) and antitumor (Qiao *et al.*, 2011) agents. They also display potential applications as corrosion inhibitors (Sauri *et al.*, 2009) and catalysts (Tamami & Ghasemi, 2011). The title molecule, (I), is a Schiff base derived from DL-phenylalanine and 2,4-dihydroxybenzaldehyde using the procedure reported by Bahron *et al.* (2010).  It crystallizes as a zwitterion in which the imine N is protonated.  A similar zwitterionic structure was reported by Hemamalini & Fun (2011).

The asymmetric unit of the title compound is shown in Fig. 1. The molecule is a zwitterion in the crystal, with the carboxylic acid group deprotonated and the imine N atom protonated. It adopts an *E* configuration about the central C9═N1 double bond [1.3045 (3) Å] with the torsion angle C10-C9-N1-C8 = -175.36 (7)°. The dihedral angle between the benzene (C10–C15) and phenyl (C1–C6) rings is 46.34 (4)°.

In the crystal structure (Fig. 2), an intramolecular N1—H1N1···O1 hydrogen bond (Table 1) generates an *S*(6) ring motif (Bernstein *et al.*, 1995). Furthermore, adjacent molecules are connected by intermolecular O1—H1O1···O4, O2—H1O2···O3, C5—H5A···O2  and C12—H12A···O2 (Table 1) hydrogen bonds forming supramolecular ribbons along the *a* axis.

### Experimental

DL-phenylalanine (2 mmol, 0.330 g ) was dissolved in absolute ethanol (20 mL), into which 2,4-dihydroxybenzaldehyde (2 mmol, 0.276 g) was added, followed by refluxing for two hours. The solution was left to slowly cool to room temperature upon which pale orange crystals were produced. These were filtered off, washed with ice-cold ethanol and air dried (yield 68%).  Melting point: 540–543 K.  Analytical calculated for C_16_H_15_NO_4_ (%): C, 67.36; H, 5.30; N, 4.91.  Found (%): C, 67.13; H, 5.49; N, 4.89. IR (cm^-1^): ν(C═N) 1642.9 (m), ν(C–OH) 1243.7 (w), ν(C═O) 1880.6 (w).

### Refinement

Atoms H1O1, H1N1 and H1O2 were located from a difference Fourier map and refined freely [N–H = 0.864 (16)Å; O–H = 0.863 (15)–1.025 (19)Å]. The remaining H atoms were positioned geometrically [C–H = 0.95–1.0 Å ] and were refined using a riding model, with *U*_iso_(H) = 1.2 *U*_eq_(C).

### Crystal data

**Table:**

C_16_H_15_NO_4_	*F*(000) = 600
*M**_r_* = 285.29	*D*_x_ = 1.413 Mg m^−^^3^
Monoclinic, *P*2_1_/*c*	Mo *K*α radiation, λ = 0.71073 Å
Hall symbol: -P 2ybc	Cell parameters from 9091 reflections
*a* = 9.3943 (1) Å	θ = 3.0–35.3°
*b* = 6.8946 (1) Å	µ = 0.10 mm^−^^1^
*c* = 20.7251 (3) Å	*T* = 100 K
β = 92.065 (1)°	Block, orange
*V* = 1341.49 (3) Å^3^	0.34 × 0.27 × 0.17 mm
*Z* = 4	

### Data collection

**Table:**

Bruker SMART APEXII CCD area-detector diffractometer	6007 independent reflections
Radiation source: fine-focus sealed tube	4783 reflections with *I* > 2σ(*I*)
graphite	*R*_int_ = 0.025
φ and ω scans	θ_max_ = 35.3°, θ_min_ = 2.0°
Absorption correction: multi-scan (*SADABS*; Bruker, 2009)	*h* = −15→13
*T*_min_ = 0.966, *T*_max_ = 0.982	*k* = −8→11
22479 measured reflections	*l* = −33→32

### Refinement

**Table:**

Refinement on *F*^2^	Primary atom site location: structure-invariant direct methods
Least-squares matrix: full	Secondary atom site location: difference Fourier map
*R*[*F*^2^ > 2σ(*F*^2^)] = 0.043	Hydrogen site location: inferred from neighbouring sites
*wR*(*F*^2^) = 0.125	H atoms treated by a mixture of independent and constrained refinement
*S* = 1.03	*w* = 1/[σ^2^(*F*_o_^2^) + (0.0674*P*)^2^ + 0.2835*P*] where *P* = (*F*_o_^2^ + 2*F*_c_^2^)/3
6007 reflections	(Δ/σ)_max_ < 0.001
202 parameters	Δρ_max_ = 0.56 e Å^−^^3^
0 restraints	Δρ_min_ = −0.24 e Å^−^^3^

### Special details

**Table:**

Geometry. All s.u.'s (except the s.u. in the dihedral angle between two l.s. planes) are estimated using the full covariance matrix. The cell s.u.'s are taken into account individually in the estimation of s.u.'s in distances, angles and torsion angles; correlations between s.u.'s in cell parameters are only used when they are defined by crystal symmetry. An approximate (isotropic) treatment of cell s.u.'s is used for estimating s.u.'s involving l.s. planes.
Refinement. Refinement of F^2^ against ALL reflections. The weighted R-factor wR and goodness of fit S are based on F^2^, conventional R-factors R are based on F, with F set to zero for negative F^2^. The threshold expression of F^2^ > 2σ(F^2^) is used only for calculating R-factors(gt) etc. and is not relevant to the choice of reflections for refinement. R-factors based on F^2^ are statistically about twice as large as those based on F, and R- factors based on ALL data will be even larger.

### Fractional atomic coordinates and isotropic or equivalent isotropic displacement parameters (Å^2^)

**Table:**

	*x*	*y*	*z*	*U*_iso_*/*U*_eq_	
O1	0.60361 (6)	0.80079 (9)	0.04375 (3)	0.01400 (12)	
O2	0.97519 (6)	0.33854 (10)	0.05402 (3)	0.01685 (13)	
O3	0.03702 (7)	0.98745 (10)	0.07713 (4)	0.01988 (14)	
O4	0.26610 (7)	1.04016 (9)	0.05323 (3)	0.01783 (13)	
N1	0.34885 (7)	0.69321 (10)	0.08580 (3)	0.01219 (12)	
C1	0.33058 (10)	0.53563 (14)	0.25232 (4)	0.01942 (16)	
H1A	0.3954	0.6401	0.2590	0.023*	
C2	0.35367 (11)	0.36077 (15)	0.28493 (5)	0.02434 (19)	
H2A	0.4337	0.3473	0.3139	0.029*	
C3	0.26067 (12)	0.20648 (14)	0.27537 (5)	0.02416 (19)	
H3A	0.2770	0.0875	0.2975	0.029*	
C4	0.14316 (11)	0.22715 (13)	0.23307 (5)	0.02018 (17)	
H4A	0.0792	0.1219	0.2262	0.024*	
C5	0.11937 (9)	0.40174 (13)	0.20089 (4)	0.01695 (15)	
H5A	0.0386	0.4150	0.1723	0.020*	
C6	0.21269 (9)	0.55797 (12)	0.20999 (4)	0.01422 (14)	
C7	0.18457 (9)	0.74664 (12)	0.17511 (4)	0.01563 (15)	
H7A	0.2505	0.8462	0.1933	0.019*	
H7B	0.0863	0.7891	0.1836	0.019*	
C8	0.20180 (8)	0.73766 (12)	0.10126 (4)	0.01250 (13)	
H8A	0.1359	0.6381	0.0817	0.015*	
C9	0.40809 (8)	0.52254 (11)	0.09102 (4)	0.01214 (13)	
H9A	0.3492	0.4173	0.1029	0.015*	
C10	0.55370 (8)	0.48144 (11)	0.08037 (4)	0.01121 (13)	
C11	0.65246 (8)	0.61982 (11)	0.05775 (4)	0.01132 (13)	
C12	0.79342 (8)	0.56952 (12)	0.04987 (4)	0.01286 (14)	
H12A	0.8591	0.6634	0.0354	0.015*	
C13	0.83916 (8)	0.37933 (12)	0.06334 (4)	0.01261 (14)	
C14	0.74286 (8)	0.23932 (12)	0.08580 (4)	0.01396 (14)	
H14A	0.7742	0.1113	0.0955	0.017*	
C15	0.60318 (8)	0.29109 (12)	0.09343 (4)	0.01367 (14)	
H15A	0.5381	0.1965	0.1079	0.016*	
C16	0.16518 (8)	0.93989 (12)	0.07349 (4)	0.01381 (14)	
H1O1	0.6599 (16)	0.855 (2)	0.0171 (7)	0.030 (4)*	
H1N1	0.3969 (16)	0.791 (2)	0.0723 (7)	0.033 (4)*	
H1O2	0.999 (2)	0.196 (3)	0.0635 (8)	0.052 (5)*	

### Atomic displacement parameters (Å^2^)

**Table:**

	*U*^11^	*U*^22^	*U*^33^	*U*^12^	*U*^13^	*U*^23^
O1	0.0131 (2)	0.0096 (2)	0.0195 (3)	0.00209 (19)	0.0036 (2)	0.0038 (2)
O2	0.0113 (2)	0.0159 (3)	0.0236 (3)	0.0048 (2)	0.0034 (2)	0.0040 (2)
O3	0.0129 (3)	0.0170 (3)	0.0299 (3)	0.0054 (2)	0.0033 (2)	0.0064 (2)
O4	0.0165 (3)	0.0150 (3)	0.0223 (3)	0.0018 (2)	0.0054 (2)	0.0063 (2)
N1	0.0109 (3)	0.0108 (3)	0.0150 (3)	0.0021 (2)	0.0019 (2)	0.0017 (2)
C1	0.0211 (4)	0.0189 (4)	0.0182 (4)	0.0017 (3)	−0.0009 (3)	0.0003 (3)
C2	0.0292 (5)	0.0244 (4)	0.0191 (4)	0.0075 (4)	−0.0044 (3)	0.0027 (3)
C3	0.0371 (5)	0.0170 (4)	0.0186 (4)	0.0083 (4)	0.0039 (4)	0.0047 (3)
C4	0.0267 (4)	0.0136 (3)	0.0207 (4)	0.0006 (3)	0.0076 (3)	0.0013 (3)
C5	0.0179 (4)	0.0155 (3)	0.0176 (4)	0.0011 (3)	0.0031 (3)	0.0011 (3)
C6	0.0165 (3)	0.0127 (3)	0.0137 (3)	0.0024 (3)	0.0043 (3)	0.0011 (3)
C7	0.0192 (4)	0.0125 (3)	0.0156 (3)	0.0028 (3)	0.0048 (3)	0.0018 (3)
C8	0.0104 (3)	0.0113 (3)	0.0160 (3)	0.0027 (2)	0.0022 (2)	0.0023 (2)
C9	0.0118 (3)	0.0110 (3)	0.0137 (3)	0.0016 (2)	0.0013 (2)	0.0010 (2)
C10	0.0102 (3)	0.0100 (3)	0.0135 (3)	0.0013 (2)	0.0013 (2)	0.0009 (2)
C11	0.0118 (3)	0.0098 (3)	0.0123 (3)	0.0016 (2)	0.0009 (2)	0.0002 (2)
C12	0.0108 (3)	0.0117 (3)	0.0162 (3)	0.0016 (2)	0.0015 (2)	0.0011 (3)
C13	0.0114 (3)	0.0129 (3)	0.0136 (3)	0.0029 (2)	0.0003 (2)	0.0007 (2)
C14	0.0129 (3)	0.0108 (3)	0.0182 (3)	0.0027 (2)	0.0014 (3)	0.0020 (3)
C15	0.0125 (3)	0.0107 (3)	0.0179 (3)	0.0013 (2)	0.0021 (2)	0.0022 (3)
C16	0.0136 (3)	0.0122 (3)	0.0157 (3)	0.0028 (2)	0.0017 (3)	0.0029 (3)

### Geometric parameters (Å, °)

**Table:**

O1—C11	1.3572 (9)	C5—H5A	0.9500
O1—H1O1	0.863 (15)	C6—C7	1.5068 (12)
O2—C13	1.3293 (10)	C7—C8	1.5461 (12)
O2—H1O2	1.025 (19)	C7—H7A	0.9900
O3—C16	1.2527 (10)	C7—H7B	0.9900
O4—C16	1.2577 (10)	C8—C16	1.5425 (11)
N1—C9	1.3045 (10)	C8—H8A	1.0000
N1—C8	1.4617 (10)	C9—C10	1.4220 (11)
N1—H1N1	0.864 (16)	C9—H9A	0.9500
C1—C2	1.3952 (13)	C10—C15	1.4152 (11)
C1—C6	1.3965 (12)	C10—C11	1.4220 (11)
C1—H1A	0.9500	C11—C12	1.3842 (11)
C2—C3	1.3863 (16)	C12—C13	1.4048 (11)
C2—H2A	0.9500	C12—H12A	0.9500
C3—C4	1.3921 (15)	C13—C14	1.4133 (11)
C3—H3A	0.9500	C14—C15	1.3745 (11)
C4—C5	1.3903 (13)	C14—H14A	0.9500
C4—H4A	0.9500	C15—H15A	0.9500
C5—C6	1.3973 (12)		
			
C11—O1—H1O1	108.9 (10)	N1—C8—C7	111.03 (6)
C13—O2—H1O2	112.1 (10)	C16—C8—C7	107.65 (6)
C9—N1—C8	125.08 (7)	N1—C8—H8A	110.1
C9—N1—H1N1	120.3 (10)	C16—C8—H8A	110.1
C8—N1—H1N1	114.6 (10)	C7—C8—H8A	110.1
C2—C1—C6	120.37 (9)	N1—C9—C10	125.23 (7)
C2—C1—H1A	119.8	N1—C9—H9A	117.4
C6—C1—H1A	119.8	C10—C9—H9A	117.4
C3—C2—C1	120.51 (9)	C15—C10—C9	117.79 (7)
C3—C2—H2A	119.7	C15—C10—C11	118.13 (7)
C1—C2—H2A	119.7	C9—C10—C11	124.07 (7)
C2—C3—C4	119.53 (9)	O1—C11—C12	121.51 (7)
C2—C3—H3A	120.2	O1—C11—C10	117.88 (7)
C4—C3—H3A	120.2	C12—C11—C10	120.60 (7)
C5—C4—C3	120.06 (9)	C11—C12—C13	119.84 (7)
C5—C4—H4A	120.0	C11—C12—H12A	120.1
C3—C4—H4A	120.0	C13—C12—H12A	120.1
C4—C5—C6	120.90 (8)	O2—C13—C12	117.22 (7)
C4—C5—H5A	119.6	O2—C13—C14	122.24 (7)
C6—C5—H5A	119.6	C12—C13—C14	120.54 (7)
C1—C6—C5	118.64 (8)	C15—C14—C13	119.06 (7)
C1—C6—C7	121.20 (8)	C15—C14—H14A	120.5
C5—C6—C7	120.17 (8)	C13—C14—H14A	120.5
C6—C7—C8	114.68 (7)	C14—C15—C10	121.81 (7)
C6—C7—H7A	108.6	C14—C15—H15A	119.1
C8—C7—H7A	108.6	C10—C15—H15A	119.1
C6—C7—H7B	108.6	O3—C16—O4	127.87 (8)
C8—C7—H7B	108.6	O3—C16—C8	114.59 (7)
H7A—C7—H7B	107.6	O4—C16—C8	117.43 (7)
N1—C8—C16	107.91 (6)		
			
C6—C1—C2—C3	0.53 (15)	C15—C10—C11—O1	178.38 (7)
C1—C2—C3—C4	−0.27 (15)	C9—C10—C11—O1	−1.95 (12)
C2—C3—C4—C5	−0.22 (15)	C15—C10—C11—C12	−1.04 (11)
C3—C4—C5—C6	0.45 (14)	C9—C10—C11—C12	178.63 (8)
C2—C1—C6—C5	−0.29 (13)	O1—C11—C12—C13	−178.40 (7)
C2—C1—C6—C7	179.02 (8)	C10—C11—C12—C13	1.00 (12)
C4—C5—C6—C1	−0.19 (13)	C11—C12—C13—O2	179.06 (7)
C4—C5—C6—C7	−179.51 (8)	C11—C12—C13—C14	−0.93 (12)
C1—C6—C7—C8	112.09 (9)	O2—C13—C14—C15	−179.07 (8)
C5—C6—C7—C8	−68.61 (10)	C12—C13—C14—C15	0.92 (12)
C9—N1—C8—C16	−166.85 (7)	C13—C14—C15—C10	−0.99 (13)
C9—N1—C8—C7	75.39 (10)	C9—C10—C15—C14	−178.64 (8)
C6—C7—C8—N1	−63.66 (9)	C11—C10—C15—C14	1.05 (12)
C6—C7—C8—C16	178.43 (7)	N1—C8—C16—O3	172.58 (7)
C8—N1—C9—C10	−175.36 (7)	C7—C8—C16—O3	−67.51 (9)
N1—C9—C10—C15	174.78 (8)	N1—C8—C16—O4	−10.87 (10)
N1—C9—C10—C11	−4.90 (13)	C7—C8—C16—O4	109.04 (8)

### Hydrogen-bond geometry (Å, °)

**Table:**

*D*—H···*A*	*D*—H	H···*A*	*D*···*A*	*D*—H···*A*
O1—H1O1···O4^i^	0.863 (15)	1.790 (14)	2.6302 (9)	163.9 (15)
N1—H1N1···O1	0.864 (14)	2.051 (15)	2.6810 (9)	129.1 (12)
O2—H1O2···O3^ii^	1.03 (2)	1.51 (2)	2.5310 (10)	179 (2)
C5—H5A···O2^iii^	0.95	2.56	3.3155 (10)	137
C12—H12A···O2^iv^	0.95	2.46	3.1781 (10)	132

Symmetry codes: (i) −*x*+1, −*y*+2, −*z*; (ii) *x*+1, *y*−1, *z*; (iii) *x*−1, *y*, *z*; (iv) −*x*+2, −*y*+1, −*z*.
